# Cases of a Disease Which Has Lately Prevailed in Some Parts of the Northern States of America

**Published:** 1815-01

**Authors:** J. Jackson, William Gamage


*For the Medical and Physical Journal.*

### *Cases of a Disease which has lately prevailed in some Parts of the Northern States of America.*

*Case by* J. Jackson, M.D.

FEBRUARY 22, 1813.—I was called to visit a truckman who was said to have the cramp in his stomach. I found him delirious and in violent distress. His face was very highly coloured, almost livid, his skin mottled in some parts, his extremities cold, pulse 74, small and frequent. Some of the time he was lying curled up on his side, and some of the time he was on his feet and endeavouring to get away, from which he was prevented with difficulty by two stout men. He said that he had been cruelly beaten in the stomach by some ruffians, and threatened revenge. I placed my hand lightly on the epigastric region, and he complained bitterly of the pain, saying, it was there he had been beaten. He had some slight convulsive affections over the whole body while I sat by him, and the attendants stated that he had had several similar affections since his seizure.

From the attendants I learnt that he had eaten his dinner as usual, and left the house apparently in perfect health. He went to his stable, and, just as he reached the door, he complained of violent distress at the stomach, which those about him supposed to be the cramp. This distress was so great that that he was unable to stand up; and immediately he became delirious. He was carried home, where he had been two hours when I visited him. During this time he had appeared constantly to be in extreme agony in the abdomen, and had vomited three times so as to throw off his dinner.

While I was examining and making inquiries into the case, about twenty minutes after first feeling his pulse, I found them very greatly altered. They were not increased in frequency, but were dilated and stronger, and had become hard.

I resolved to perform venesection; but this was difficult on account of the constant agitation and delirium of the patient. He submitted, with reluctance, partly to force and partly to persuasion. The blood spouted freely from a large orifice, but his restlessness caused the bleeding to be irregular and slow. The difficulty diminished remarkably during the flow of blood. After four or five ounces were taken, he began to grow more calm; after ten or twelve ounces his face became more pale; before he had lost sixteen ounces he fell asleep, and while the blood was still flowing in a small stream he awoke perfectly rational. After the loss of eighteen ounces of blood the pulse were 66, but not diminished in strength nor in fulness.

The patient now confirmed the account which I had received of his seizure. He said that the attack of pain in the epigastric region was extremely sudden and severe, that it continued to be very distressing with great soreness, that it was increased by the least motion and by an attempt to gape or to make a full inspiration. His head and limbs were also very painful.

In the remaining history of this case I need not be minute, as the circumstances already stated show its character. Twelve more ounces of blood were taken after three hours, and now as before a thick inflammatory crust was formed. He was blistered over the epigastrium, and the next day he was freely purged. He took submuriate of quicksilver with tartrite of antimony and opium. On the second day the disease was fast subsiding. On the third day he walked about the room, and on both these days he coughed and expectorated thick mucus with ease. On the fourth day he went abroad.

### *Cases by* William Gamage, jun. M.D.

Case 1st.—In the evening of January 27th, 1813, I saw Mrs. G. She had considerable difficulty of breathing, and complained of distress over the whole chest, without adverting to any one part as being more painful than another; but, when asked, she said, it was most acute in the right side.

She

She had pain in her head, back, and limbs; skin hot, but moist; tongue coated white; pulse feeble, quick, and sharp. The disease commenced suddenly with a severe chill, and she had been ill about eighteen hours. I directed a cathartic, and, after its operation, a pill, consisting of two grains of calomel, one of opium, and one of one quarter of a grain of tartrite of antimony.

In the morning her symptoms were not mitigated, though the medicines had operated, as expected. I determined to bleed her, and took away, by a large orifice, sixteen ounces of blood; her pulse immediately became fuller and soft. The blood on standing exhibited a pretty thick buff. The pain was much relieved, except in the right side, where it was felt more acutely. A blister was applied to this side, and the pill, the opium being diminished to a quarter of a grain, given every eight hours; mild drinks indulged in freely.

29th.—The blister had produced its effect, but the pain in the head and side and some difficulty of breathing, still remained; she had a frequent and hard cough; pulse quick and hard. I again opened a vein, and took twelve ounces of blood, and the pulse became soft. After this bleeding the disease seemed to be abating, till the evening of the 31st, when from some imprudence the pain in the side, distress, and difficulty of breathing, returned with great violence; pulse small, quick, and hard. I immediately bled her to the amount of sixteen ounces, and relief soon followed this evacuation. The next day the mouth was sensibly affected by the calomel; but it was some time before her cure was complete. She was young, and had rather a delicate constitution.

Case 2d.—February 12th, 1813. I was called towards evening to Mr. E. B., a stout hard-working man, of about thirty-five. He was suffering with very difficult respiration, violent pain in the right side, and distress across the lower part of the thorax; pain in the head, back, and limbs; hot but moist skin; tongue coated white; countenance flushed and distressed; pulse small and quick, and easily lost by pressing on the artery. The disease commenced with a severe chill, and had continued about twelve hours. I immediately bled him from a large orifice to the amount of from 26 to 30 ounces. Before I had finished, he expressed his joy for the relief which it gave him, and begged me to take more. He was not in the least faint, and his pulse in a few minutes became large and full. The blood exhibited a very thick buff. A cathartic was directed, and mild drinks.

During the night the bandage on the arm from which he was bled got loose, and he lost by this accident, as I judged, about about six or eight ounces of blood. This was followed by no disadvantage; the pain in the side was still acute; respiration difficult, and the pulse quick and hard, and seemed to demand the evacuation even more than before the first operation; I therefore took from the same orifice, eighteen hours after the first bleeding, sixteen ounces, and the pulse became slower and soft. The blood exhibited much less buff than yesterday. A blister was applied to the afflicted side, and the submuriate of quicksilver was given, as in the former case. Assisted by one or two gentle cathartics, he recovered in a few days so as to take tonic medicines.

Case 3d.—March, 1813. De G., a common labourer, and a very robust man, had a severe ague fit, which was followed by great distress in the region of the diaphragm, and pain in the left side; skin hot, and the pulse quick and small; was relieved by one copious bleeding immediately after the attack. Assisted by a blister, cathartic, and calomel, he got well in a short time.

Case 4th.—March 6th. E. S., a domestic servant, after a chill was seized with violent pain in her right side and shoulder, inability to move her right arm, and difficult respiration, pain in the head and limbs; pulse small and quick, and easily compressed; skin hot, tongue coated. I took from her in a large stream twenty ounces of blood, and she was relieved. The bandage which was applied to the arm getting in the night above the opening in the vein, caused a considerable loss of blood; the exact amount, however, could not be ascertained. The accident was entirely in her favour. She recovered rapidly with very little medicine.

Case 5th.—Mrs. C., a labouring woman, had symptoms very similar to those in the 4th case. She got well soon after one copious bleeding.

Case 6th.—March 31st. Mr. R., intemperate in his habits, and between fifty and sixty, was seized with the most excruciating pain in the right hypochondrium, accompanied with difficult respiration. He took a large dose of senna and manna, which operated smartly, but produced not the least abatement of the pain. He became delirious; skin hot, but in a profuse perspiration; pulse small and quick; but I bled him to the amount of twenty-four or twenty-six ounces, and the pulse became slow and full. The pain gradually subsided, and he was well in a few days.

Case 7th.—In the evening of April 9th, 1813, I visited Bloke, a young man, and a sailor. He was prostrate in his bed, and breathing with much difficulty; had great distress in the chest, particularly about the heart; violent pain in his head and limbs, and a total inability to move the right arm or or leg. He went to bed the evening before apparently well, and was taken in the night; he had been ill about sixteen hours, and much of the time delirious; skin hot; pulse small and quick. I determined to bleed him; and watching the pulse, I perceived, that when I had taken about a pint of blood, it became large and tense; I let it run, till I had obtained above half a pint more, and the pulse seemed soft. After this he was rational, and his pains much abated. He then took an emetico-cathartic, which operated smartly, and he could move his limbs. The submuriate of quicksilver in small doses was directed; in three days it affected his mouth. He recovered without further difficulty.

Several instances of slighter affections of the lungs occurred during this period, in which bleeding was employed with happy effects. In fact, the lancet has been a very useful companion to the materia medica in most of the varieties of disease of the latter part of winter and the spring.

I had no opportunity of observing after death the morbid effects of this disease in any case, where the symptoms had previously been under my own inspection. By the politeness of friends, I have been present at three or four examinations of those who died by this disease. The phenomena exhibited on those occasions, the hardness of some part of the lungs, their gorged state, their dark colour, approaching almost to black, the pericardium red and thickened, or covered with livid spots, and other marks of the most violent inflammation, demonstrated to me, in a manner the most positive, the necessity of copious evacuations of blood to cure this disease, notwithstanding the forbidding aspect of the pulse, which commonly attends it. I was indebted to an early opportunity of this kind for, at least, my confidence in bleeding as a principal remedy; and, so far as I have known, every case in which this remedy had not been used freely and early in the attack, has terminated unfortunately.—*New-Eng. Journal.*

